# Spawning of Bluefin Tuna in the Black Sea: Historical Evidence, Environmental Constraints and Population Plasticity

**DOI:** 10.1371/journal.pone.0039998

**Published:** 2012-07-24

**Authors:** Brian R. MacKenzie, Patrizio Mariani

**Affiliations:** 1 Center for Macroecology, Evolution and Climate, National Institute for Aquatic Resources (DTU Aqua), Technical University of Denmark, Charlottenlund, Denmark; 2 Center for Ocean Life, National Institute for Aquatic Resources (DTU Aqua), Technical University of Denmark, Charlottenlund, Denmark; National Oceanic and Atmospheric Administration/National Marine Fisheries Service/Southwest Fisheries Science Center, United States of America

## Abstract

The lucrative and highly migratory Atlantic bluefin tuna, *Thunnus thynnus* (Linnaeus 1758*;* Scombridae), used to be distributed widely throughout the north Atlantic Ocean, Mediterranean Sea and Black Sea. Its migrations have supported sustainable fisheries and impacted local cultures since antiquity, but its biogeographic range has contracted since the 1950s. Most recently, the species disappeared from the Black Sea in the late 1980s and has not yet recovered. Reasons for the Black Sea disappearance, and the species-wide range contraction, are unclear. However bluefin tuna formerly foraged and possibly spawned in the Black Sea. Loss of a locally-reproducing population would represent a decline in population richness, and an increase in species vulnerability to perturbations such as exploitation and environmental change. Here we identify the main genetic and phenotypic adaptations that the population must have (had) in order to reproduce successfully in the specific hydrographic (estuarine) conditions of the Black Sea. By comparing hydrographic conditions in spawning areas of the three species of bluefin tunas, and applying a mechanistic model of egg buoyancy and sinking rate, we show that reproduction in the Black Sea must have required specific adaptations of egg buoyancy, fertilisation and development for reproductive success. Such adaptations by local populations of marine fish species spawning in estuarine areas are common as is evident from a meta-analysis of egg buoyancy data from 16 species of fish. We conclude that these adaptations would have been necessary for successful local reproduction by bluefin tuna in the Black Sea, and that a locally-adapted reproducing population may have disappeared. Recovery of bluefin tuna in the Black Sea, either for spawning or foraging, will occur fastest if any remaining locally adapted individuals are allowed to survive, and by conservation and recovery of depleted Mediterranean populations which could through time re-establish local Black Sea spawning and foraging.

## Introduction

Many marine fish species such as anchovy, *Engraulis encrasicolus*, and cod, *Gadus morhua,* are distributed over wide geographic ranges which include both fully marine and estuarine habitats. Estuarine-spawning populations of marine fish species usually have evolved physiologies and life histories to enable successful establishment, including reproduction, in such habitats. In this study, we consider the possibility that a large pelagic highly migratory species, bluefin tuna, has also evolved such adaptations.

Bluefin tuna used to be seasonally common in the Black Sea [1]–[3] whose salinity is only half that of the Mediterranean Sea. The species used to migrate there from the Mediterranean for feeding but no bluefin tuna has been seen or captured in the Black Sea since the late 1980s [4], [5]. The species is now believed to be absent from the area, but the reasons for its disappearance are unclear. Explanations in the literature include overexploitation, shipping noise and changed environmental conditions (e.g., eutrophication); however the mechanisms by which these factors might have affected bluefin tuna are obscure, largely undocumented and unquantified [1], [4], [6]. The Black Sea disappearance is part of a contraction of the overall range of the species since the 1950s [7], [8].

Nevertheless, and regardless of the causes of the disappearance of this population, the historical ecology of this species in the Black Sea is poorly understood, and represents a challenge to our knowledge of the life history and dynamics of this species. Its presence there is most thoroughly documented based on commercial fishery data and sightings, but abundances, distributions, and biological rates (e. g., growth) have never been quantified [1], [2]. Lack of knowledge of its life history and ecological role in this ecosystem could inhibit the development of new science-based attempts to manage a reappearance and recovery of this population. For example, the loss of a local population may represent a decrease in the population richness and thus intra-specific biodiversity of this species.

This would be the case if the species migrated to the Black Sea for feeding and probably also for reproduction [1], [2]. Occupation of specific habitats for reproduction, and the subsequent development of offspring in isolation from those produced in other areas, is believed to be a mechanism which can lead to the development of distinctive populations, including those having specific genetic characteristics [9]. In the context of this paper, we consider a population as a locally-reproducing group of individuals with distinguishable characteristics and traits, and which occupies a specific area across generations for reproduction [9].

Examples of marine fish species containing genetically distinct populations in different habitats are numerous [10] and include cod on specific banks on the continental shelf off eastern Canada [11] and several species across the salinity gradient from the North Sea into the Baltic Sea [12]. Atlantic bluefin tuna also has significant genetic population structuring throughout its range and at more local scales within the Mediterranean Sea [13]–[15]. Population richness, and the different traits that individual populations possess, contribute to a “portfolio effect” to promote successful reproduction throughout the species range under diverse conditions [16]–[18]. A decline or loss of a population with specific adaptations to local conditions could therefore increase the risk that the species will decline further.

**Figure 1:**
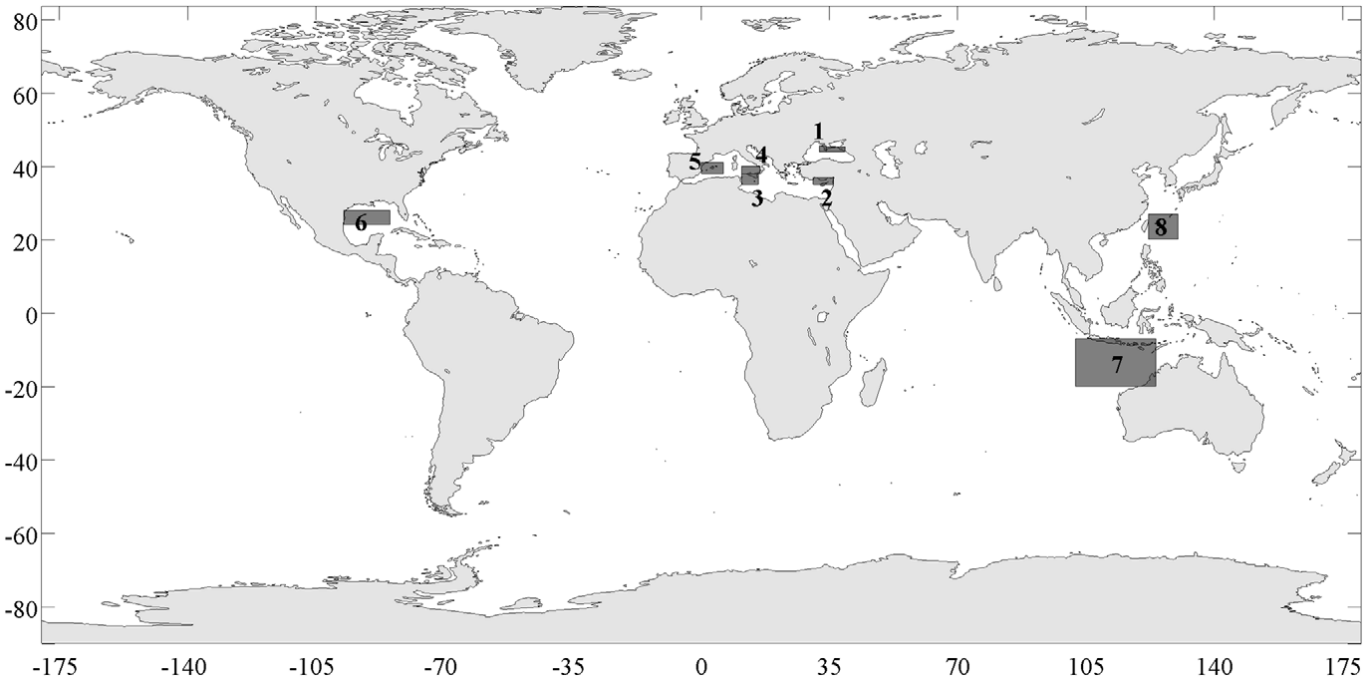
Map showing spawning areas for bluefin tuna species throughout the world, as estimated from ichthyoplankton surveys and gondal development of adults. Atlantic bluefin tuna *T. thynnus*: 1) Black Sea, 2) Levantin Sea, 3) Sicily Channel, 4) Tyrrhenian Sea, 5) Balearic sea, 6) Gulf of Mexico; southern bluefin tuna *T. maccoyii*: 7) northern Australia - Indonesia; Pacific bluefin tuna *T. orientalis*: 8) Japan Sea. Latitude and longitude coordinates for each area are available in Table 1.

In this study, we investigate the possibility that bluefin tuna used to spawn in the Black Sea, and evaluate some of the adaptations that the species must have had for this reproduction to have been successful. We focus on buoyancy – density interactions between bluefin tuna eggs and the hydrographic conditions in spawning areas. This interaction is likely critical for successful reproduction in the Black Sea because it is a relatively fresh environment with colder, anoxic water in deep layers [6]. We hypothesize that conditions in the Black Sea differ sufficiently from those in other areas where bluefin tunas spawn (Atlantic *T. thynnus,* Pacific *T. orientalis* Temminck & Schlagel 1884, and southern: *T. maccoyii* Castelnau 1872; Figure 1), that if spawning did occur in the Black Sea, the population would have been adapted to produce eggs whose buoyancy could allow eggs to survive the local hydrographic conditions.

**Table 1:** Spawning times and areas for bluefin tuna species, *T. thynnus*, *T. orientalis* and *T. macoyii*, compiled from literature.

Region and species	Time	Reference	Location
Black Sea; *T. thynnus*	June-July	[1], [19]	44–45.6 N; 32.1–34 E;44–45.33 N; 34–39.5 E
Balearic; *T. thynnus*	June-July	[1], [126];	38–41 N; 0–6 E
Levantine Sea, Cyprus; *T. thynnus*	May-June	[70], [71]	35–37 N; 30.5–36 E
Sicily Channel; *T. thynnus*	June-July	[1]	35–38 N; 11–15.5 E
Tyrrhenian Sea; *T. thynnus*	June-July	[1]	38–40 N; 11–16 E
Gulf of Mexico; *T. thynnus*	Mid-April-June	[1], [27]	24–28 N; 85–97.5 W
Western Pacific; *T. orientalis*	April – June	[28], [29], [127]	20–27 N; 122–130 E
Australia-Indonesia; *T. macoyii*	September-April	[30]	7–20 S; 102–124 E

These ideas are evaluated by deriving and comparing spawning site “climatologies” for all known areas in the global ocean where bluefin tunas spawn, comparing the sea water densities with those of bluefin tuna eggs, and modelling the sinking rates of bluefin tuna eggs spawned in the Black Sea.

## Methods

The study is based on a literature review and analysis of existing hydrographic and egg buoyancy data, and a modelling exercise. The study consists of the following sub-topics:

- evaluation and synthesis of literature evidence of spawning in the Black Sea;
- extraction, compilation and presentation of hydrographic data for bluefin tuna spawning areas around the world;
- modelling the sinking speed of bluefin tuna eggs;
- compilation of egg buoyancy data for marine fish species spawning across wide salinity ranges.

### Literature Evidence of Bluefin Tuna Spawning in the Black Sea

We compiled literature describing spawning and reproduction of bluefin tuna in the Black Sea. The literature we considered was based on ichthyoplankton, adult migration and gonadal development data. The ichthyoplankton data are summarized in reviews [1], [2], [19] and we accessed original Ukrainian and Russian language literature cited by [1], [6], [20] for further details of the findings. We also considered the migration and reproductive data that have been compiled from commercial fisheries sources [21]. The commercial catch data can provide information about the seasonality, direction and location of spawning migrations, as well as the reproductive status of individual fish (i. e., gonads in a sexually mature, but pre-spawning, ripe or spent state). In some cases when eggs were captured, authors measured and reported sizes (diameters) of eggs and the oil globule. Since marine fish species which spawn in low salinity habitats often produce larger eggs due to higher water content than populations which spawn in higher salinity environments [22]–[26], we noted and compared these sizes; we hypothesized that the bluefin tuna eggs produced in the Black Sea are (were) larger than those produced in other regions.

### Construction and Comparison of Bluefin Tuna Spawning Site Climatologies throughout the Global Ocean

We compared the long-term hydrographic conditions in bluefin tuna spawning areas in the Black Sea with those in other well-documented spawning areas of the Mediterranean Sea (Levantine Sea north of Cyprus, south of Sicily, southern Tyrrhenian Sea, south of Balearic Islands). Spawning areas and times (Figure 1, Table 1) were extracted from the literature and based on collections of eggs and larvae at sea and the appearance of spawning bluefin tuna. In addition, we included in our comparison the spawning areas for the western Atlantic population of this species located in the Gulf of Mexico [1], [27], and for the two other bluefin tuna species, *T. orientalis*, in the western Pacific near Japan [28], [29], and *T. maccoyii* (northern Australia-Indonesia; [30]).

Hydrographic data for these areas were retrieved from the World Ocean Atlas database 2009 [31] which was accessed during Nov. 2010 – July 2011 (http://www.nodc.noaa.gov/OC5/WOD09/pr_wod09.html). We used raw historical oceanographic profiles for temperature, salinity and dissolved oxygen taken by different instruments at discrete depth over the period 1880–present. A simple quality control and filtering procedure was then applied to exclude spikes and nonsense data, and to include only casts with (at least) temperature and salinity observations measured together. To exclude nonsense data (wrong depth, unphysical values, etc) specific casts showing extreme values were individually checked for consistency. This procedure excluded on average ∼3% of the data with the value for the Black Sea <<1%.

We used in the analysis only data for the upper 150 m of salinity, temperature, and oxygen concentration during the months of peak spawning (Table 1). Measures of practical salinity (psu) were converted into absolute salinity (g/kg) and *in-situ* density profiles were calculated using the international thermodynamic equation of seawater [32], [33]. Oxygen data were extracted to evaluate whether bluefin tuna eggs and larvae would encounter low oxygen concentrations at depths of neutral buoyancy. The profiles for each variable were plotted for all years to visualize their shapes; a mean profile with 95% confidence limits was estimated using General Additive Modelling as implemented in the MGCV package of R software (www.r.org), and using depth as predictor variable. The GAM analysis used a Gaussian log-link function and lowess smoother [34], [35]; degrees of freedom for smoothing were 8 for density and salinity profiles, and 4 for temperature and oxygen profiles. The GAM analyses for this investigation were only conducted to derive long-term mean profiles and confidence limits, rather than developing best-fit models and identifying sources of variation within and among areas.

### Bluefin Tuna Egg Buoyancy Data

No study has yet measured the vertical distribution and *in situ* buoyancy of bluefin tuna eggs in any of its spawning areas in the world. However, ichthyoplankton sampling and capture of bluefin tuna larvae in the Mediterranean Sea has been successful in the upper 70 m [36], and upper 70–100 m [37]. Some limited measurements of buoyancy of bluefin tuna eggs from maricultured (caged) adults in the Mediterranean are available [38], and we have used these in our analyses. However, due to the few measurements of the specific gravity of bluefin tuna eggs in the Mediterranean, we also use laboratory data for the related species Pacific bluefin tuna *T. orientalis* [39] and assume as a first-order approximation that the buoyancy of its eggs and larvae are reasonably comparable to those of *T. thynnus*.

We then considered the depths at which bluefin tuna eggs might be buoyant in the Black Sea. The depth of neutral buoyancy of a fish egg generally is a complicated function of egg size, water and dry matter content, external salinity during ovary development and hence adult blood osmality, and the possibility that adults have adapted to the local salinity conditions [23]–[25], [40]–[43]. Generally however, after the egg has been fertilized, their densities are relatively insensitive to salinity of external seawater [42], [44], [45].

In this case, it is possible to estimate the approximate depth at which bluefin tuna eggs and larvae might have been neutrally buoyant in the Black Sea during former spawning periods, if Black Sea tuna produced eggs with similar buoyancies as bluefin tuna in the Mediterranean Sea (e. g., near Cyprus or Sicily). Furthermore given those depths, it is also possible to estimate the ranges of temperature, salinity and oxygen concentration to which the eggs would have been exposed, and the consequences of that experience on successful egg fertilisation, development and hatching. Those abiotic conditions can then be compared with the hydrographic conditions in other geographic areas and with the ranges that bluefin tuna eggs and larvae need in order to develop successfully. Alternatively, and assuming that bluefin tuna did indeed spawn in the Black Sea, they may have produced eggs with lower specific gravity than in other higher salinity areas. Such eggs would have higher buoyancy in the Black Sea and would allow them to float higher in the water column. As will be shown below, fish species with geographic ranges whose spawning sites include low salinity areas often produce eggs which are more buoyant in low salinity areas than in high salinity areas. These issues will be addressed further in the Discussion.

### Modelling the Vertical Transport of Bluefin Tuna Eggs in the Black Sea

If there is a density difference between the eggs and the surrounding water, the eggs will rise or sink until they reach a depth at which they are neutrally buoyant. We estimated whether eggs would remain in the oxygenated, warm part of the water column of the Black Sea if they were spawned in the surface, and if they had a density similar to those produced by Mediterranean Sea bluefin tuna.

The vertical distribution of fish eggs and larvae can be described by a transport equation which balances particles buoyancy and vertical mixing [41], [46], [47]. Excluding the effects of mortality and the velocity of the fluid, the concentration of bluefin tuna eggs (C) in a mono-dimensional water column can be described as:
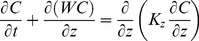
(1)which expresses the rate of change of C as function of the divergence of the eggs vertical flux [46]. The model is a two parameter advection diffusion equation where the vertical velocity (W) depends only on the buoyancy of the eggs while the diffusion coefficient (K_z_) is related to the vertical structure and mixing of the water column.

Introducing moving coordinates the convective terms in Eq. 1 can be eliminated reducing it to a simple diffusion equation (e.g. [48]). Assuming uniform and constant values for W and K_z_ and an instantaneous particle release at z = z0, an analytical solution of Eq. 1 can be obtained for an infinitive deep water column:
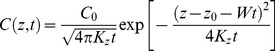
(2)


The value of W depends on the balance between gravity and drag forces around the sinking particle. Estimates of the terminal velocity, W, are mainly provided by empirical relations of particles size (S), density (ρ_S_) and shape [49] and depend on the surrounding water density (ρ_W_) as well as the hydrodynamic regime at which particles are exposed. Different expressions of W have been suggested for different ranges of Reynolds number (Re):
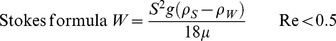
(3a)

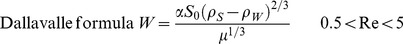
(3b)where Re  =  ρ_W_ WS/μ is the Reynolds number, μ the dynamic molecular viscosity (μ = 10^−3^ Pa s, at 20°C), g the gravitational acceleration constant (g = 9.81 m s^−2^) and α an empirical constant (α = 0.08825). Combining Eq. 3a with Re  = 0.5, the maximum size (S_max_) at which the Stokes formula holds can be derived:



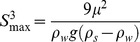
(4)Then the corrected size to be used in Eq. 3b can also be defined as S_0_ =  S − 0.4·S_max_ (e.g. [46]).

We conducted simulations using different combinations of egg densities as reported in the literature and spawning depths. Moreover, we used a mixed layer numerical ocean model [50] to simulate the dispersion of negatively buoyant particles in a water column with an initial density profile similar to that obtained for the Black Sea (Figure S1). The vertically varying velocity W(z) is dynamically calculated from Eq. 3b while the diffusivity, K_z_(z), is obtained from a k-ε turbulence model [51] imposing a light wind stress on surface (τ = 1 N m^−2^ ) and a minimum dissipation rate ε = 10^−8^ m s^−3^ (Figure S1). After 3 days of this wind, we released 50,000 numerical particles and followed them for a further 2 days using a Lagrangian particle random walk algorithm [52]. For the sake of generality we randomly released the particles between 0 and 10 m to account for a large range of bluefin tuna spawning depths. We assumed that spawning depth (i. e., depth where eggs were released from females) was located at a depth range whose temperature and oxygen conditions were most likely to promote egg fertilisation, development and hatching. Egg densities were the mean and lower limits of measured ranges.

A final model simulation was a sensitivity analysis of the time required for eggs of different densities to sink out of the upper 10 m of the water column. In this case we used both the long-term average density profile in the Black Sea and a higher density profile (1 standard deviation). This simulation was conducted to estimate the range of densities that bluefin tuna eggs should have in order to have high probability of remaining within a layer of water with sufficient oxygen and temperature conditions for successful development.

### Evaluation of Among-population Variability in Egg Density for Other Species

Many marine fish species have evolved to reproduce in continental shelf regions and estuaries where salinities are much lower than in fully marine conditions. To evaluate the range of egg specific gravities among populations of different species, and thus the possible range of adaptation to local salinities among those populations, we compiled from the literature and compared egg specific gravities for several other fish species which spawn naturally over wide ranges of salinities (e. g., cod).

Egg densities were usually measured in a density water column [53]. Eggs were usually obtained by stripping from spawning females. Density measurements were most often performed on fertilised eggs. Fertilisation was usually done at the local salinities where adults were captured. In cases where density was measured at several ages during egg development, we used only the measurements during the first 1–2 days to avoid introducing additional variability associated with ontogenetic changes in density [39], [54]. We included density measurements for unfertilized eggs since densities of fertilized and unfertilized eggs are similar during early stages of development [43], 55.

In a second group of studies, authors conducted experiments to investigate the influence of non-native salinity on egg density. One type of experiment involved capture of adults and then transfer to lower or higher salinity for several weeks, months or even 1–2 years while the ovaries developed. Eggs produced by the exposed adults were then used for density measurements. A second type of experiment involved capture of adults and fertilisation of eggs at a range of salinities, including those which were typical and atypical for the capture habitat. A third type of experimental study involved collection of eggs at sea and their transfer onboard to different salinities to estimate buoyancy responses; water of different salinity was created by dilution with freshwater or addition of sea salt.

A third group of studies which provided data to our study involved ichthyoplankton sampling of eggs from the sea. Densities of these eggs where then measured onboard in a density column.

The measured density of the eggs for a given population was then compared with the salinities at which: i) the eggs were fertilized, (ii) the adults were held during gonadal development, or iii) the eggs were captured. Egg densities for all populations of a species were plotted together vs. salinity for visual comparison. We then evaluated in a meta-analysis the general hypothesis that populations and species which spawn in waters of different salinities produce eggs whose buoyancies are positively related to local salinities. This hypothesis was evaluated using linear regression of egg buoyancy vs. local salinity for all populations and species. We conducted a second and similar meta-analysis of egg buoyancy measurements made in nonlocal salinities; these data were derived from experimental data involving transfer of adults or eggs captured *in situ* to different salinities, or fertilisation and/or subsequent incubation of eggs at nonlocal salinities. This analysis evaluated the extent to which eggs retained the density characteristics of local populations even when exposed to atypical salinity (i. e., egg buoyancy is hypothesized to be independent of the salinity experienced by transferred adults, or during fertilisation or incubation). Differences in slopes between the two egg buoyancy vs. salinity regressions therefore indicate the extent to which local populations retain their egg buoyancies when confronted with nonlocal salinities, and thus how strongly egg buoyancy can be considered to be a population-level trait.

The data we extracted from each study were group means for individual sampling sites or experimental trials within studies. In some cases, standard deviations or ranges of densities were given, and these have also been extracted and plotted to illustrate the variability in egg density within sites or treatments. The corresponding salinities were usually given as averages and ranges; in cases where eggs were sampled from the field and where salinity profiles were given in the report, we extracted the mean or range midpoint of salinities at the depths were eggs were collected. We estimated some egg buoyancies from vertical distributions of eggs within stratified water columns; in these cases we assumed that egg vertical distributions were relatively unaffected by vertical mixing processes and that mean sea water density for the depth range of capture was indicative of the density of egg neutral buoyancy. In some reports, egg vertical distributions could be extracted but no hydrographic data were provided; in these cases, we searched the literature for estimates of hydrographic conditions which could be used to derive densities using seawater equations of state, or extracted long-term mean density profiles from international hydrographic databases (e. g, ICES, World Ocean Atlas) for the season and location when/where eggs were captured.

The focus of this compilation was on the comparison of egg densities for multiple populations within species and to support our interpretation of possible adaptation to low salinity spawning in the Black Sea by bluefin tuna. The compilation is therefore indicative of biogeographic differences in egg buoyancies within and among species, although there are many more egg buoyancy data in the literature or which could be derived than are represented in our dataset. For example, we excluded egg buoyancy measurements for species where egg buoyancy data were available for only one population in one hydrographic situation, and egg buoyancy estimates from well-mixed regions (e. g., tidal zones) because of the strong role that mixing would have on egg distributions.

The entire database (Table S1) compiled from the literature is available as an online Supplement spreadsheet file or from the authors.

## Results

### Bluefin Tuna Reproduction in the Black Sea

Existing literature suggests that bluefin tuna reproduced in the Black Sea [1], [2] (Table 2). Bluefin tuna would migrate into this sea in late spring-early summer to spawn and then emigrate again in late summer and autumn. One purpose of the migration was for feeding [1], [2], [21]. However the timing of the immigration and emigration is also consistent with a spawning migration. This is supported by some limited gonadal development data collected when bluefin tuna entered and exited the Black Sea. Bluefin tuna captured while immigrating to the Black Sea contained gonads which were in a developing or near fully ripe state, whereas gonads of bluefin tuna captured late in the year during emigration were spent [1], [21].

**Table 2:** Summary of ichthyoplankton studies which have sampled bluefin tuna eggs in the Black Sea (no egg concentrations were given in the studies).

Reference	Area/region	Sampling years	Depth rangeof sampling	Presence/absence
[56]	Near Sevastopol	1933	Not stated	Present
[57]	Sampled near Karadag region (located on Crimea nearSimferopol’ and Yalta). Author states that results were similarto those seen near Sevastapol and Novorossiisk so authorconsidered results from all 3 areas to be representativefor NE part of Black Sea.	Not stated	Not stated	present
[58]	Northern Black Sea	1948–51	Not stated	present
[1], [59]	NW Black Sea (Mys Meganom andCape Tarkhankut, Ukraine)	1957	0–1 m	present
[62]	Southern Black Sea	1993	0–120 m	absent

Early ichthyoplankton sampling captured bluefin tuna eggs and larvae in the Black Sea during the 1930s-1950s [1], [19], [56]–[58]. Most of these eggs were captured in the north-central part of the Black Sea in July-August [19], [56], [58]. The sampling locations are not described in detail in this literature but most sampling was done in waters south of the Crimea peninsula as part of ichthyoplankton sampling to describe spawning times for the entire fish community and to identify species of fish which reproduced in this region. Most sampling was done within 30–100 m of the shore although some stations were located 10 nautical miles offshore [59]. It is unknown whether ichthyoplankton sampling has been conducted in this time period in the southern half of the Black Sea. Several eggs captured at sea have been incubated until hatching and up to 8 days of larval life afterwards to confirm taxonomic assignment [1], [59]. The timing of the capture of the eggs corresponds to the timing of immigration and emigration, and seasonal change in gonadal development that has been reported from historical catch data in the Sea of Marmara [1], [21]. The sampling depths were usually 0–3 m [19], [59]; for example 5 eggs were captured in the neuston (upper 1 m) in 1957 [19].

The sizes of eggs captured in the Black Sea were ca. 0.98–1.10 mm (Figure S2), and mean size of the oil globule was 0.28 mm. Egg sizes of naturally spawning bluefin tuna in other regions are not available for comparison. However, bluefin tuna eggs collected from various sea-ranching operations in the Mediterranean or Japan in which adults were fed artificial diets and sometimes hormonally-stimulated to spawn produced eggs which were somewhat smaller [60] or approximately the same size [38], [61].

The identification of eggs captured in the earlier studies has been questioned by some later investigators [36], [62]. An ichthyoplankton survey in the Black Sea for bluefin tuna eggs and larvae in the early 1990s failed to capture any specimens; however by this time, bluefin tuna had already disappeared from the region [4], [5].

### Geographical Comparison of Vertical Profiles of Hydrographic Variables in Bluefin Tuna Spawning Areas

**Figure 2:**
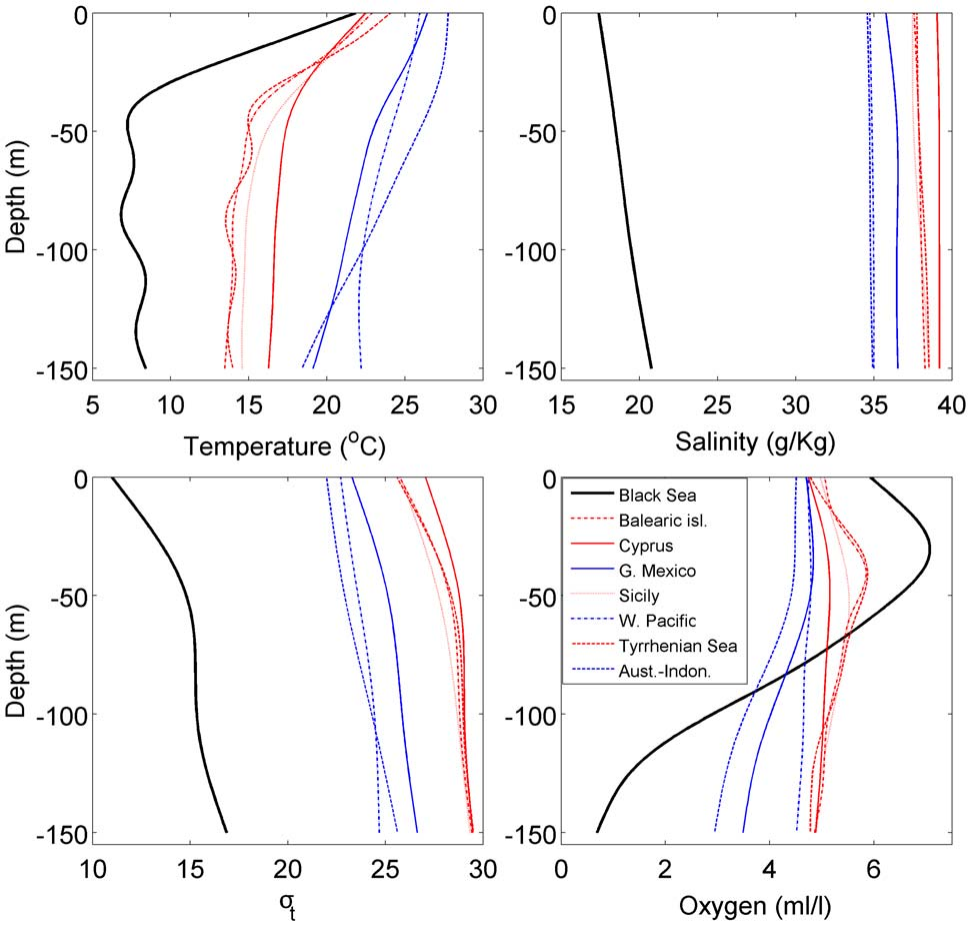
Long-term averaged vertical profiles of density, salinity, temperature and oxygen concentration in bluefin tuna spawning habitats (Black Sea, Levantine Sea, south of Sicily, southern Tyrrhenian Sea, south of Balearic Islands, Gulf of Mexico, Japan, northern Australia - Indonesia) during peak spawning periods (**Table 1**) in different regions. Data source [31]. Each line is a statistical fit to 100s of data points using General Additive Models (Table S2). See all observed data with GAM fits and 95% confidence intervals in site-specific vertical profiles for each variable in Supporting Information (Figures S3, S4, S5, S6).

Vertical profiles of temperature, salinity, oxygen concentration and density for the different areas where bluefin tuna spawn in the global ocean show some difference among areas, and as expected the Black Sea differs most (Figure 2; Figures S3, S4, S5, S6). In most areas, temperature in the upper 30 m is ca. 23–26°C, salinity is 36–38 g kg^−1^, and dissolved oxygen concentration is 4–5 ml l^−1^. However regional differences are evident.

Temperatures in Mediterranean spawning habitats are ca. 3–5°C lower in the upper 40–50 m than in the Gulf of Mexico, west Pacific and Austral-Indonesian areas, notably reflecting the latitudinal gradient from more temperate to more tropical areas. Black Sea temperatures are the lowest among all bluefin tuna spawning habitats. The vertical gradient of temperature was greatest in the Black Sea, followed by Mediterranean sites and the remaining sites. Notably, in the Black Sea, there is a strong decline of temperature from ca. 23 to 12°C throughout the upper 40 m, after which temperatures are nearly constant to 150 m.

Salinity conditions also differed among areas in a pattern analogous to that seen for temperature. The Mediterranean (excl. Black Sea) salinities as a group were all higher than other spawning areas; differences were ca. 2–4 g kg^−1^ depending on which areas are compared. In all areas, salinity was homogeneous with depth. The Black Sea salinity profile differed substantially and was much lower (ca. 50% of salinities in other areas); its vertical shape also differed because salinities gradually increased with increasing depth (Figure 2, S2).

Due to the higher salinity in the Mediterranean areas (range 37.3–38.8 g kg^−1^ compared with 35–36.5 g kg^−1^ in other areas), Mediterranean density profiles were also on average higher than profiles from other areas. The Black Sea density profile differed most from all other areas; the Black Sea density range is 1011.5–1017 kg m^−3^, whereas densities in all other areas are >1022 g kg^−1^ at all depths considered here. As the salinity profiles in all these areas are nearly vertically homogeneous, vertical differences in density are primarily due to thermal differences among regions.

Oxygen conditions at the surface (upper 5–10 m) in all areas except the Black Sea were similar, but profiles through the water column differed. Oxygen conditions increased with depth in the Mediterranean and then decline, but generally remain higher by 1–2 ml l^−1^ at all depths than concentrations in the other 3 areas. The Black Sea oxygen profile differs greatly from all other areas. Oxygen concentration in the Black Sea surface layer is highest among all areas and increases down to ca. 60 m; concentrations then decline and with depth until 125 m and reach hypoxic levels (≤2 ml l^−1^).

As we are primarily interested in long-term conditions we focussed on the mean shapes and magnitudes of the profiles. However inter-annual and other sources (e. g., within-region spatial) of variability is evident in the profiles. This variability is seen in the 95% confidence limits provided for all profiles in all regions in Supporting Information figures.

**Figure 3:**
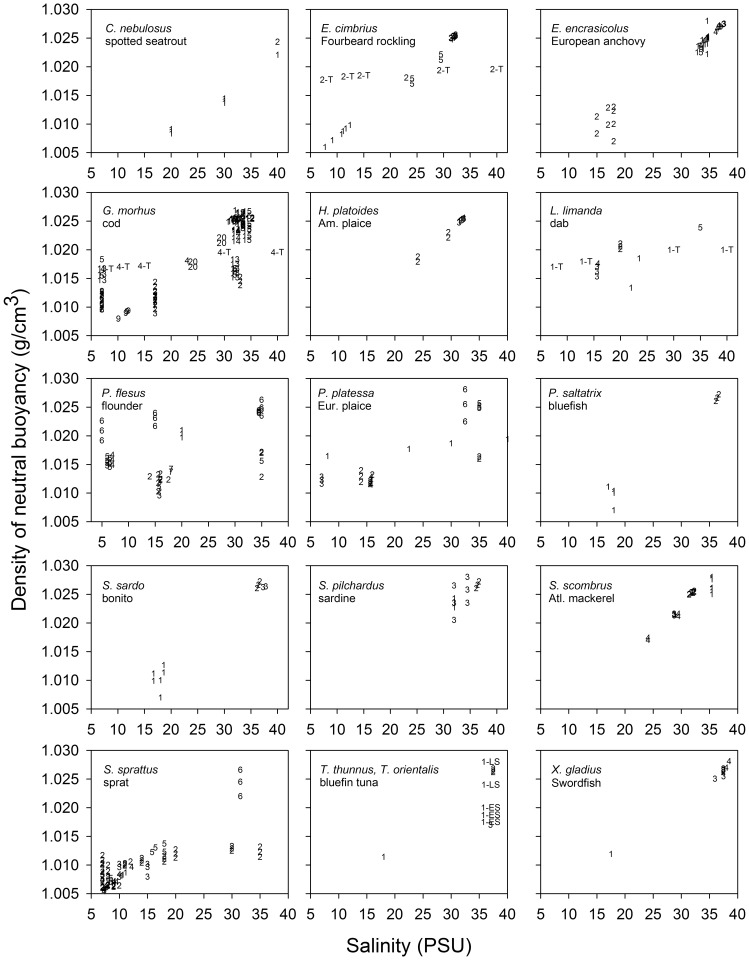
Seawater density of neutral buoyancy for populations of 16 marine fish species inhabiting habitats with different salinities, compared with the natural local salinity at fertilisation or capture. Fish eggs for most populations and species were obtained by stripping eggs and fertilisation in the laboratory or onboard research vessels (denoted as lab-fertilised below) or were captured at sea in ichthyoplankton surveys (denoted as field-captured below). One study [44] involved capture of live eggs and transfer to different salinities for buoyancy measurements; numerical codes for these data are shown with a “T” in panels. Symbols represent different populations within following species: *Cynoscion nebulosus*, spotted seatrout (lab-fertilised) [69]: 1– Matagorda Bay, Texas; 2– Upper Laguna Madre, Texas; *Enchelyopus cimbrius,* fourbeard rockling (all eggs captured at sea): 1 =  Baltic Sea, Gotland Basin [104], 2 =  Baltic Sea, Kiel Bay (field-captured and transfered) [44], 3 =  Conception Bay, Newfoundland, Canada [105], 4 =  Placentia Bay, Newfoundland, Canada [106] with hydrographic data from [107], 5 =  Tracadie Bay offshore, Gulf of St. Lawrence, Canada [108]; *Engraulis encrasicolus,* anchovy, (all eggs captured at sea): 1– Bay of Biscay [109], [110]; 2– Black Sea [20], [111] with hydrographic data from [112], 3 - Gulf of Lyons [54]; 4– NW Africa-Morocco [113], 5– Po River plume, northern Adriatic Sea [114]; *Gadus morhua,* cod: 1– Arcto-Norwegian cod: Lofoten (lab-fertilised) [25]; 2 - eastern Baltic (lab-fertilised) [24], [26]; 3– Baltic Sea, Gotland Basin (lab-fertilised) [25], [26]); 4 and 4-T –Baltic Sea, Kiel Bay (field-captured and transferred) [44], 5– Baltic Sea, ICES SD 23 (lab-fertilised) [26], 6– Baltic Sea, ICES SD 24 (lab-fertilised) [26], 7– Baltic Sea ICES SD 25 (lab-fertilised) [26], 9– Baltic Sea, ICES SD 26 (field-captured) [104], 10– Conception Bay, Newfoundland, Canada (field-captured) [105], 11– Gulf of St. Lawrence, Canada (field-captured) [115], 12– Grand Banks, Newfoundland, Canada (field-fertilised) [116], 13– Gullmarenfjord, Kattegat, western Sweden (lab-fertilised) [26], 14 - inshore Newfoundland, Canada (lab-fertilised) [116], 15– Norwegian coastal cod (lab-fertilised) [23]; 16– Norwegian coastal cod, Helgeland, Norway (field-captured) [117], 17- Norwegian coastal cod, Øygården, Norway (field-captured) [117], 18 - Norwegian coastal cod, Porsanger, Norway (field-captured) [117]; 19– Norwegian coastal cod, Tysfjord, Norway (field-captured) [117], 20– Tracadie Bay offshore, Gulf of St. Lawrence, Canada (field-captured) [108]; *Hippoglossoides platessoides,* American plaice: 1– Conception Bay, Newfoundland, Canada (field-captured) [105], 2 - Tracadie Bay offshore, Gulf of St. Lawrence, Canada (field-captured) [108], 3– Trinity Bay, Newfoundland, Canada (field-captured) [118]; *Limanda limanda,* dab: 1– Baltic Sea, Kiel Bay (field-captured and transferred) [44] and (lab-fertilised) [40], 2– Baltic Sea, ICES SD 23 (lab-fertilised) [43], 3 Baltic Sea, ICES SD 24 (lab-fertilised) [43]; 4 - Baltic Sea, ICES SD 25 (lab-fertilised) [43]; 5– Bergen, Norway (lab-fertilised) [40]; *Platichthys flesus*, flounder (all are lab-fertilised except eggs captured at sea at site 7): 1– Baltic Sea, ICES SD 23 [43], 2 - Baltic Sea, ICES SD 24 [22], [40], [43], 3 - Baltic Sea, ICES SD 25 [43], 4 - Baltic Sea, ICES SD 28 [43], 5– Baltic Sea, Tvärminne, Finland [22], [40], [45]; 6 -Bergen,Norway [22], [45]; 7 - Black Sea [20] with temperature data from [6]; *Pleuronectes platessa*, European plaice: 1–Baltic Sea, Kiel Bay (field-captured and transferred) [44], 2- Baltic Sea, ICES SD 24 (lab-fertilised) [22], [43]; 3 - Baltic Sea, ICES SD 24–25 (lab-fertilised) [43], 4 - Baltic Sea, ICES SD 25 (lab-fertilised) [43]; 5- Bergen, Norway (lab-fertilised) [22]; 6– North Sea, southern (field-captured) [119]; *Pomatus saltatrix*, bluefish (all are field-captured): 1– Black Sea [20], [66] with temperature data from [112], 2 =  NW Africa, Morocco [120]; *Sarda sarda*, bonito: 1– Black Sea (field-captured) [20], [121], 2 =  NW Africa-Morocco (field-captured) [113], 3 =  NW Mediterranean-Spain (lab-fertilised in land-based tanks) [38]; *Sardina pilchardus*, sardine (all are field-captured): 1– Bay of Biscay [109]; 2– NW Africa, Morocco [113]; 3 - Plymouth, UK [68]; *Scomber scombrus*, Atlantic mackerel: 1– Celtic Plateau (field captured and lab-fertilized) [53], 2– Conception Bay, Newfoundland, Canada (field captured) [105], 3 - St. George’s Bay, so. Gulf of St. Lawrence, Canada (field captured) [122], 4– Tracadie Bay offshore, Gulf of St. Lawrence, Canada (field captured) [108]; *Sprattus sprattus,* sprat: 1– Baltic Sea, Gotland Basin (field captured) [104], 2 - Baltic Sea, SD 25 (lab fertilised) [84], [92], 3 - Baltic Sea, ICES SD 25–28 [92], 4– Baltic Sea, SD 26 (field captured and lab fertilised) [92], [104], 5– Black Sea (field captured) [20], [67] with temperature data from [112], 6– Plymouth, UK (field captured) [68]; *Thunnus thynnus*, Atlantic bluefin tuna and *Thunnus orientalis*, Pacific bluefin tuna: 1 - unknown developmental stages of *T. thynnus* from the Black Sea caught in the upper 1 m of the water column [19], 2– northern Ionian Sea and Strait of Messina, Mediterranean Sea [63] (field-captured) 3 - unknown developmental stages of *T. thynnus* from the northwest Mediterranean (lab-fertilized) [38]; 1-ES and 1-LS – early and late-stages of *T. orientalis* eggs collected in *in situ* rearing cages in Japan [39]; *Xiphias gladius*, swordfish (all field captured): 1 - Black Sea [89], 2– Ionian Sea and Strait of Messina, Mediterranean Sea ([63], 2– Mediterranean Sea, 3 - so. Tyrrhenian Sea [123], 4– Mediterranean Sea, NW Aegean Sea [124], with hydrography data from [125].

**Figure 4:**
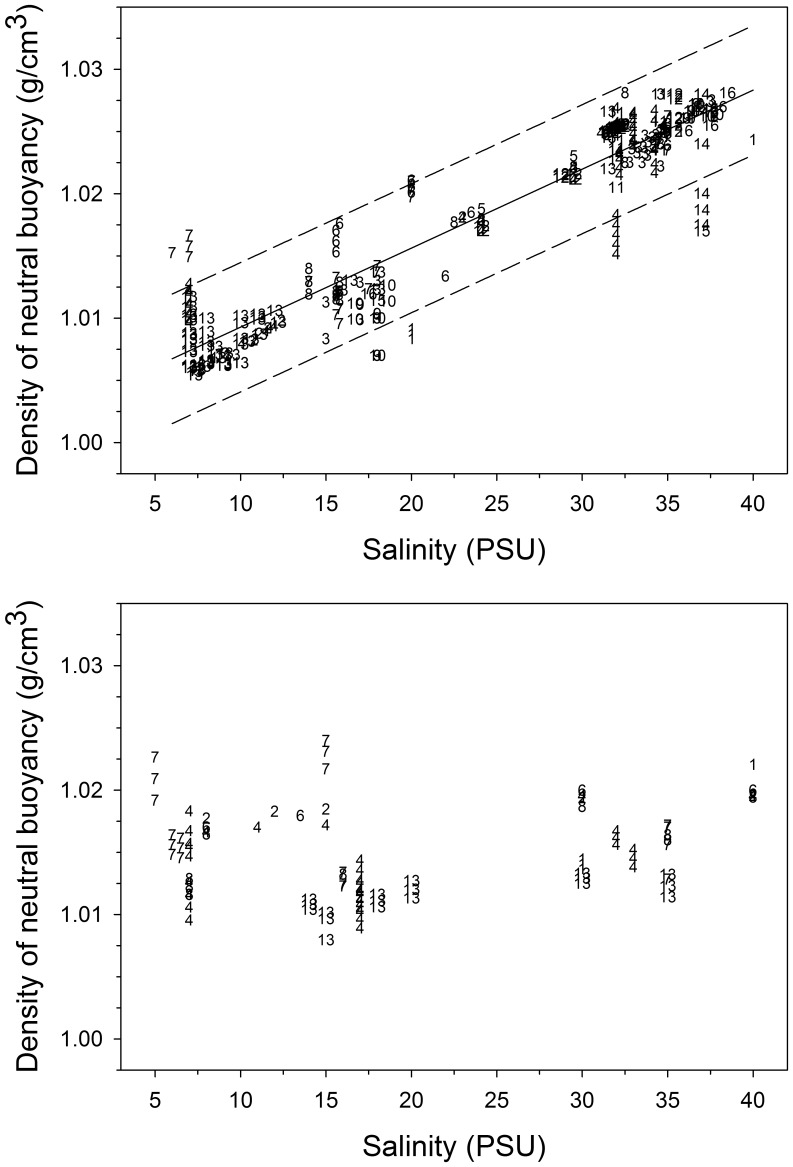
Top panel: Density of neutral buoyancy of eggs from 16 species of fish in relation to the salinity of water during gonadal maturation, egg fertilisation and egg incubation in local spawning areas. Solid line: linear regression model; dashed lines: 95% prediction limits. Regression statistics: y = 0.0009*x+1.0029; R^2^_adj._ = 0.87; P<0.0001; residual mean square error SD_est_  = 0.0026; N = 336. Species codes: 1 =  *Cynoscion nebulosus* spotted seatrout, 2 =  *Enchelyopus cimbrius* fourbeard rockling, 3 =  *Engraulis encrasicolus* European anchovy, 4 =  *Gadus morhua* cod, 5 =  *Hippoglossoides platessoides* American plaice, 6 =  *Limanda limanda* dab, 7 =  *Platichthys flesus* flounder, 8 =  *Pleuronectes platessa* European plaice, 9 =  *Pomatus saltatrix* bluefish, 10 =  *Sarda sarda* bonito, 11 =  *Sardina pilchardus* sardine, 12 =  *Scomber scombrus* Atlantic mackerel, 13 =  *Sprattus sprattus* sprat, 14 =  *Thunnus orientalis* Pacific bluefin tuna, 15 =  *Thunnus thynnus* Atlantic bluefin tuna, 16 =  *Xiphias gladius* swordfish. Bottom panel: same as top panel, except that salinities were atypical of those in local spawning areas because adults were transferred to nonlocal salinities for gonadal development, spawning and fertilisation, eggs were fertilised and/or incubated at nonlocal salinities, or eggs were captured at sea and then transferred to nonlocal salinities for buoyancy measurements. The relationship is not statistically significant (P = 0.14; N = 99). Species codes (N = 7) as above. Populations and data sources given in Figure 3 and Supplementary Table 1.

The density of Atlantic bluefin tuna *T. thunnus* eggs (Figure 3, 4) spawned in captivity by adults from the northwest Mediterranean Sea is 1017 kg m^−3^ [38]. The variability or range of the reported density was not reported, and no information about ontogenetic changes in egg density was presented, nor was the stage of development of the eggs used for density measurements stated. The reported density is at the lower limit of the range of density (1018–1020 kg m^−3^) measured for eggs of Pacific bluefin tuna *T. orientalis* in early stages of development [39], and produced by adult Mediterranean bluefin tuna fed artificial diets in a sea-ranching operation; as eggs approached hatching, density increased to 1020–1028 kg m^−3^. Densities of bluefin tuna eggs captured in the upper 25 m of the Ionian Sea, Mediterranean Sea [63] can be estimated to be 1026–1027 kg m^−3^ (Figure 3, 4).

### Modelled Vertical Distribution of Bluefin Tuna Eggs in the Black Sea

Bluefin tuna eggs are typically S = 1 mm in diameter (e.g. [60], [64]) and measurements of specific gravity [38], [39], [65] provide density estimates typically ranging between 1017 kg m^−3^ and 1020 kg m^−3^ (Figure 3); we initially use the range midpoint, ρ_S_  = 1019 kg m^−3^ from the experimental measurements in simulations, although field sampling suggests that bluefin tuna eggs from the Mediterranean are much denser than this range [63]. Our data on the summer water density of the Black Sea range between ρ_w_  = 1011–1017 kg m^−3^ (Figure 2) yielding S_max_ between 0.5–0.8 mm which indicates that Eq. 3b should be applied in this case. Hence the terminal velocity relevant for sinking of bluefin tuna eggs in the Black Sea is W  = 1–3 mm s^−1^.

**Figure 5:**
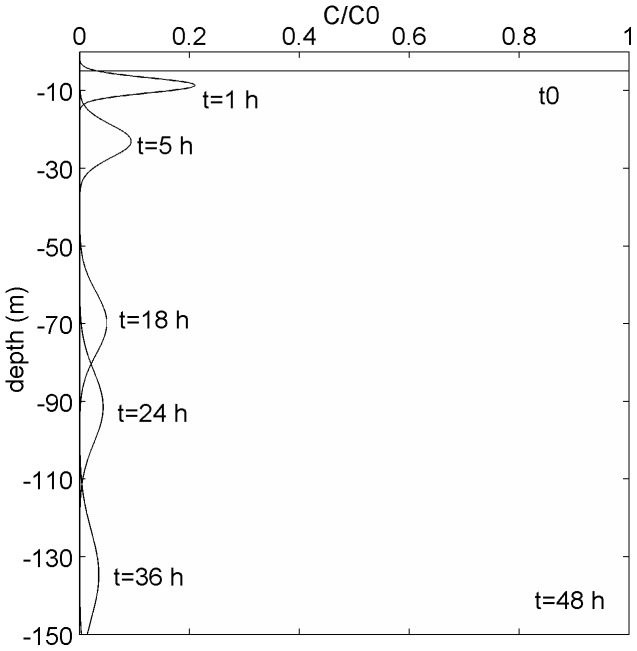
Analytical solution of the distribution of sinking bluefin tuna eggs in the Black Sea over time (0–48h). Particles have been released at time t0 and depth z0 = 5 m with an initial concentration C_0_ and terminal velocity W  = 1 mm s^−1^, corresponding to egg density  = 1019 kg m^−3^.

Solving Eq. 2 for a single instantaneous release at z0 = 5 m in a relatively low turbulent environment (K_z_ = 5×10^−4^ m^2^ s^−1^) and with a conservative value of terminal velocity W  = 1 mm s^−1^ we find that most of the eggs sink below 50 m after only 18 hours and they reach the bottom (150 m) after 48 h (Figure 5). On the other hand, at the lower end of egg density (ρ_S_ = 1017 kg m^−3^), the eggs are neutrally buoyant at the depth of 150 m for the long-term averaged profile (Figure S3), while they are neutrally buoyant at 115 m and 65 m when the 68% and 95% confidence intervals are considered.

**Figure 6:**
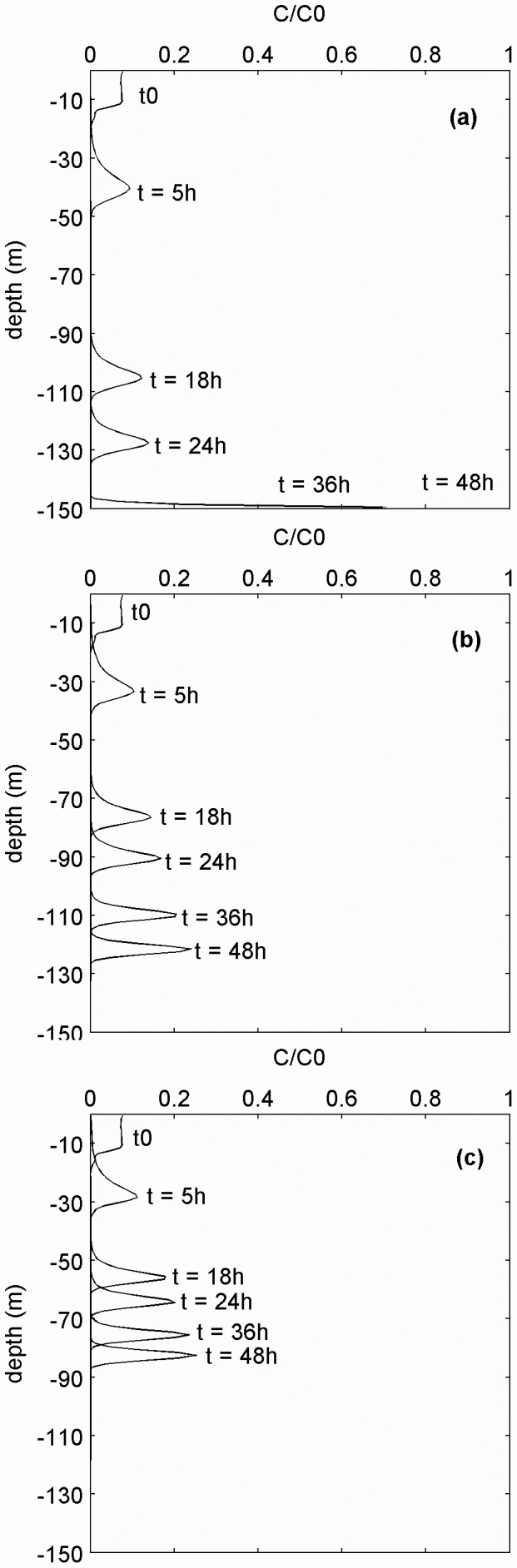
Simulated vertical distribution of bluefin tuna eggs at different times and specific gravities under modest wind-induced mixing. Panels from top to bottom represent simulations for egg densities ρ_S_  = 1019, 1017 and 1015.7 kg m^−3^. Particle concentration calculated from the average number of particles passing the numerical box of 1 m side between two time steps (dt = 60 seconds).

We tested unusual density conditions (using the mean +1 SD density profile) and the effects on egg dispersion with a more realistic numerical model, where wind mixing, turbulence diffusion and egg buoyancy are directly estimated from first principles (Figure 6). Using ρ_S_  = 1019 kg m^−3^, after 5 h, most particles are distributed at 30–50 m and after 18 h at 90–110 m while >55% accumulated at the bottom after 32 hours (Figure 6a). There are no eggs suspended in the water column after 38 h or in the first 10 m (the spawning interval depth) after 9 h.

Assuming egg density ρ_S_  = 1017 kg m^−3^, all the eggs sink from the upper 10 m after 10 h and are distributed within a narrow range at 120 m depth after 48 h (Figure 6b). Finally, assuming that the variability (i. e., range) of density of Mediterranean bluefin tuna eggs was similar to that for early-stage Pacific bluefin tuna eggs (difference between minimum and maximum  = 0.0025) and that the mean is approximately the range midpoint, then an estimate for the lower range of egg density for Mediterranean bluefin tuna would be 1017–2.5/2 = 1015.7 kg m^−3^. When eggs having this density throughout ontogeny are released in the upper 10 m, all eggs sink out of the upper 10 m after 19 h (Figure 6c).

**Figure 7:**
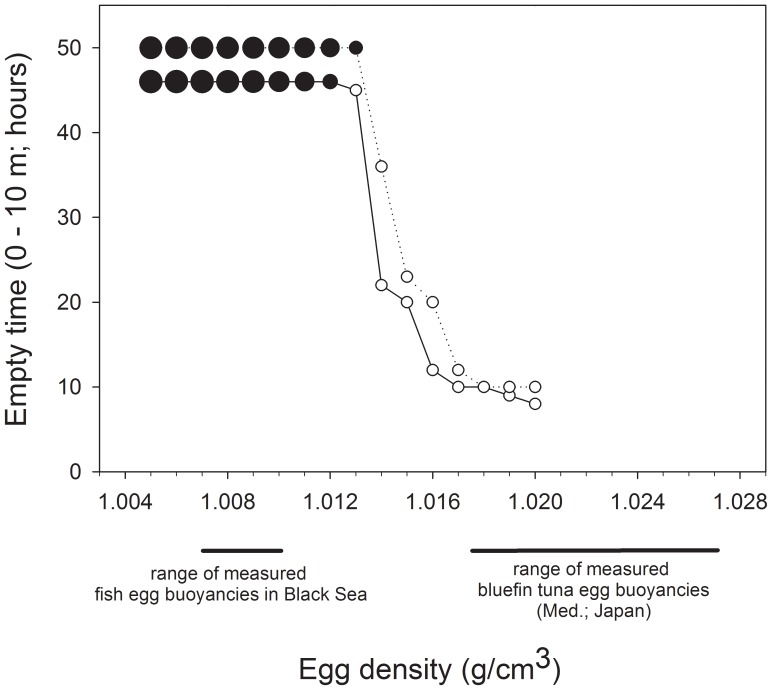
Time after which no bluefin tuna eggs remain in the upper 10 m of the numerical domain vs. egg density. For densities ρ_S_ ≤1012 kg m^−3^ some eggs can remain within the upper 10 m during 48 h, even under modest wind-induced mixing. The proportion of eggs remaining is linearly scaled to symbol size for the black circles; white circles indicate 0% of eggs remaining in upper 10 m. Solid and dashed lines represent simulations assuming long-term mean and mean +1 SD of density profile as estimated by GAM of World Ocean Atlas data. The solid and dashed lines for densities ρ_S_ ≤1012 kg m^−3^ are shown plotted at 48−2 and 48+2 hours for clarity. Horizontal bars below figure indicate ranges of measured buoyancies of fish eggs in the Black Sea [20] and for bluefin tuna eggs in the Mediterranean Sea and near Japan.

The sensitivity analysis of egg sinking time for different egg densities showed that a large fraction of eggs with densities ≤1012 kg m^−3^ could remain within the upper 10 m of the water column for sufficiently long period to hatch at local temperatures (Figure 7). Eggs with slightly higher densities (1013 kg m^−3^) might also experience sufficient temperature in some years (given the inter-annual variability in historical data; Figure S5), but eggs with progressively higher densities will sink to depths where temperatures and oxygen concentrations (Figures 3, S5, S6) would inhibit development. However bluefin tuna eggs with such low densities have not been reported, while densities in the range 1007–1010 kg m^−3^ have been measured [20] (Figure 7) and can be inferred from depth-specific sampling of many other fish species spawning in the Black Sea [66], [67].

### Among-population Variability in Egg Density for Other Species

Egg density data were found for multiple populations of 16 species (Figure 3, 4). These data show two broad patterns. First, densities of neutral buoyancy for eggs measured in “native” (local) salinities for the population which provided the eggs (i. e., salinity at fertilisation similar to the salinity usually experienced in nature by the adults during gonadal and egg development) usually increased with the salinity at fertilisation. This pattern is evident for most species for which measurements from several populations are available (e. g., cod, sprat, anchovy). For example, cod eggs produced and fertilized in the eastern Baltic Sea (salinity range 7–15 PSU) have lower densities of neutral buoyancy than cod eggs from higher saline regions such as the Skagerrak, Norwegian fjords or on Grand Bank, Newfoundland, Canada (Figure 3, 4). Similarly, anchovy eggs captured at sea in different salinities within a region (e. g., Bay of Biscay) and or across regions show an increase in density of neutral buoyancy as salinity increases (Figure 3, 4). Meta-analysis linear regression showed that density of neutral buoyancy significantly increased with local salinity (Figure 4) for all populations and species considered in this analysis.

However a second pattern shows that if eggs from a given population are fertilised at atypical (non-local) salinities or if adults are transferred to non-local salinities for gonadal and egg development, the eggs usually retain neutral buoyancy which is nearly characteristic of their local salinity. For example, when cod eggs from the eastern Baltic Sea (local salinity  =  ca. 7 PSU) were fertilized and incubated at 15 or 35 PSU, their neutral buoyancies were nearly the same as for eggs measured at 7 PSU (Figure 3, 4). Similar and even more striking patterns are evident for Baltic sprat, whose eggs retain a neutral buoyancy density typical for the Baltic salinity range 7–15 PSU even when exposed to salinity  = 20–35 PSU (Figure 3, 4). In contrast, sprat eggs captured at sea in fully marine conditions (English Channel; [68]) had a much higher density of neutral buoyancy (Figure 3, 4). Moreover, sprat eggs have been captured at sea in the upper 3 m of the Black Sea water column [20], where salinity was probably ca. 18 PSU (Figure 2); sprat egg density in the Black Sea was therefore also considerably lower than in fully marine situations.

A large number of transfer experiments with Baltic flatfish species (flounder, plaice, dab) show the adaptability of egg buoyancy to local conditions [22], [43]. For example, exposure of Baltic adult flounder or plaice to higher salinities has relatively little influence on density of neutral buoyancy for the eggs (Figure 3, 4). Similarly, if flounder from a high salinity location (35 PSU) are exposed to lower salinities (15 or 5 PSU), the density of neutral buoyancy still remains much higher than for eggs produced by Baltic flounder at the same low salinities (Figure 4). Spotted seatrout from two estuaries with different salinities also showed similar patterns in transfer experiments [69]: the population exposed to lower salinity still produced eggs of lower density of neutral buoyancy than the population adapted to higher salinity when both populations were exposed to high salinity (Figure 3, 4). Lastly, exposure of sea-captured eggs in the western Baltic Sea to nonlocal salinities [44] had relatively little influence on egg buoyancy among four fish species (Figure 3).

Bluefin tuna egg buoyancies have only been measured at one salinity (37 PSU) and there are few depth-stratified collections of bluefin tuna eggs in the literature [63]; comparisons among populations are therefore limited by data availability. However Zaitsev’s (1959) collection of bluefin tuna eggs in the upper 1 m of the Black Sea suggests high buoyancy; given the long-term mean temperature and salinity measured there (22°C; 17.5 PSU; Figure 3), these eggs would have had a neutral buoyancy of 1011 kg m^−3^, and therefore been considerably more buoyant than in other spawning areas (Figure 3, 4).

These results for individual species with multiple populations are general across all species and populations considered. Meta-analysis linear regression of the density of neutral buoyancy vs. salinity for measurements conducted at nonlocal salinities showed that egg density varied independently of the experimental salinity (P = 0.14; Figure 4).

## Discussion

### A Comparative Climatology of Bluefin Tuna Spawning Habitats around the World

This analysis and synthesis of hydrographic data includes all the known spawning areas for bluefin tunas. However the full spatial extent of spawning may perhaps not yet be known, as shown by recent discoveries of new spawning areas. For example, the spawning area for *T. thynnus* near Cyprus has only been confirmed in the early 2000s [70], [71]. More recent ichthyoplankton sampling (2009) indicates that Atlantic bluefin tuna probably also spawns in the western Carribbean Sea [72]. Moreover, data-storage tagging studies indicate that some adult Atlantic bluefin tuna do not occupy the known spawning areas at times of year corresponding to known spawning times [73], [74]. These observations suggest that bluefin tuna might be using presently undocumented spawning areas or times, or skipping spawning in some years [74]. In addition, spawning areas used could perhaps change during the lifetime of the species [71]. These observations indicate that the full extent and fidelity of habitats presently and formerly occupied by spawning bluefin tuna are still uncertain (at least for *T. thynnus*), and that our knowledge of reproductive biology, including hydrographic conditions experienced by eggs and larvae, in the wild is incomplete.

Long-term mean hydrographic conditions in spawning areas are one of many factors which shape population and species’ life histories, including spawning and migration behaviour and phenology. The results presented here show some systematic differences among some of the areas. In the Mediterranean Sea (excluding Black Sea), mean temperature, salinity, density and oxygen profiles are nearly identical, and differences are generally small. However as a group the Mediterranean conditions differ from those in the Gulf of Mexico, the western Pacific (near Taiwan and south Japan), and the waters between Australia and Indonesia. As such, they may provide first-order baselines of mean hydrographic conditions against which future changes due to for example climate change [27], [28] could be evaluated for specific populations or in a comparative manner across populations and species.

The present profiles may also be useful when comparing some key physiological rates, life history traits (e. g., spawning time, migration behaviour) and genetic patterns among the species and populations. This approach has been insightful for studies of the macroecology of other fish species, notably small pelagic clupeids (e. g., anchovy, sardine; [75], [76]), and cod ([77], [78]). In the case of bluefin tuna, one might hypothesize that among-population variations in reproductive and early developmental processes could be related to the differences in hydrographic conditions at spawning sites and nursery areas. Bluefin tuna sampled on spawning areas in the Gulf of Mexico and Mediterranean Sea during spawning time differ genetically [14]. Moreover tagging and otolith microchemistry data of adult bluefin tuna across several years suggest exclusive spawning site preference. Indeed individuals visiting the Gulf of Mexico (or Mediterranean) spawning areas during known spawning periods have never been observed visiting Mediterrenean (or Gulf of Mexico) areas during the same period in other years [79]–[81]. The modest difference in hydrographic conditions between these areas may have contributed to the evolution of such differences. There are also genetic differences among populations within the Mediterranean Sea [15], where hydrographic conditions among known spawning areas are smaller than those between the Mediterranean and the Gulf of Mexico. Hence processes and mechanisms in addition to abiotic conditions at spawning sites also likely contribute to the local structuring of bluefin tuna populations (e. g., food and predator abundances; advective processes).

Given these profiles, the salinity and thermal conditions for reproduction in the Black Sea (Figure 2) appear initially to be restrictive for bluefin tuna in this system. Salinities are ca. 50% lower than in other spawning areas and could be too restrictive for successful fertilisation and development of eggs. However, other marine fish species spawn and reproduce successfully in estuarine regions [24], [26], [45], [82], [83] so we cannot presently exclude the possibility that bluefin tuna could do (or did) the same. This issue is discussed in a following section.

Regarding temperature, bluefin tuna eggs require 19–20°C to develop and hatch in the laboratory [38]. Temperatures in the upper 20–30 m layer of the Black Sea often reached and exceeded this range (Figure S5), which would be warm enough to allow many bluefin tuna eggs to develop and hatch. However, *average* temperature conditions in the Black Sea decrease more sharply with depth than in other areas where bluefin tuna are known to spawn: mean temperature at 5 m depth is already below 20°C in the Black Sea (Figure 2). Nevertheless, the presence of variability suggests that in *some* years and places within this region there is likely sufficiently warm conditions for the eggs to develop and hatch successfully. Moreover some bluefin tuna used to spawn in August in the Black Sea [1], and conditions at this time were likely warmer than in June. Had (or if) there are still bluefin tuna spawning in the Black Sea, new experimental studies could (should) be conducted to evaluate the thermal and salinity requirements for egg fertilisation and development.

Our perception of the spawning area in the Black Sea is limited by fragmentary descriptions of the sampling locations for eggs and larvae, and is constrained to where eggs have been captured by ichthyoplankton surveys. These surveys have mostly been conducted in the northern area near Crimea, until a survey in the 1990s was conducted in the south-central region; however by this time, the population had disappeared. The true extent of where bluefin tuna used to spawn in the Black Sea is unknown. It is probable that other (southern) areas of the Black Sea are warmer than areas considered here, and possible that bluefin tuna may have spawned in those locations. Consequently our hydrographic representation of bluefin tuna spawning habitat in the Black Sea is perhaps an underestimate due to limited sampling and knowledge of spawning locations.

### Density Constraints on Reproduction of Bluefin Tuna in the Black Sea

Our analyses and comparisons show that bluefin tuna from the Mediterranean Sea are unlikely to produce eggs which could remain buoyant in the upper layers of the Black Sea where temperature and oxygen conditions are suitable for successful egg development. Eggs produced by these spawners, as well as by a closely-related species *T. orientalis*, are too dense relative to the hydrographic conditions of the Black Sea to remain within the warm, oxygenated surface layer. Our modelling exercise showed that such eggs would sink into cold anoxic waters within 5–10 hours. This time period is shorter than the expected egg development time (24–40 hours [38], [61] estimated for 21–27°C). If sinking time was sufficiently slow, the newly hatched larvae might be able to swim up to surface layers and avoid detrimental conditions at depth. Hatch success of bluefin tuna eggs decreases strongly at temperatures <20°C and >29°C [38], [61]. Temperatures which allow bluefin tuna eggs to hatch in the Black Sea are only found in the upper 10–20 m (Figure 2, S5). Sinking of bluefin tuna eggs to colder water would therefore delay and probably prevent development. Moreover sinking of eggs to depths >50–60 m would expose the eggs to oxygen concentrations <2–3 ml l^−1^. Given that bluefin tuna eggs in other areas occupy surface oxygen-rich areas, it is most likely that oxygen concentrations <3 ml l^−1^ will also inhibit normal development and hatch success. Consequently the contemporary Black Sea is unlikely to be a suitable habitat for most bluefin tuna eggs from the Mediterranean Sea.

Alternatively, one could hypothesize that the bluefin tuna population which may have spawned in the Black Sea produced eggs which were adapted to the specific conditions of this ecosystem, including having a density of neutral buoyancy which was sufficiently low to maintain the eggs in the warm, oxic layer at the surface. Such an adaptation would allow eggs to experience temperature and oxygen conditions comparable to those in other bluefin tuna spawning areas throughout the world. Notably bluefin tuna eggs have been captured in the upper 1 m of the Black Sea [19]. These eggs must have been considerably more buoyant than eggs of contemporary bluefin tuna spawning in the Mediterranean Sea (Figure 4 and Figure 7).

There is another possibility which could explain the presence of bluefin tuna eggs in the Black Sea. As suggested by one of our reviewers, Black Sea spawners could possibly have been a “sink” population from the Mediterranean, and produced eggs there without successful fertilisation and/or development. These eggs could potentially have been captured by ichthyoplankton sampling before sinking from the upper layer of the water column. We cannot exclude this possibility of “sink spawning” although given the rapid egg development time and sinking time (if not adapted to local hydrographic conditions), we suspect that such captures would have been unlikely.

Many other marine fish species which spawn in estuarine ecosystems produce buoyant eggs to enable flotation in lower density habitats (Figure 3, 4); moreover, reciprocal transfer experiments and other experiments where eggs are exposed to a wide range of non-local salinities shows that this ability is a population-level trait [24], [26], . Indeed, meta-analysis showed that egg density in such experiments varied independently of the experimental salinity treatment, whereas egg densities measured at typical, local salinities were strongly correlated with those local salinities. Egg characteristics which promote higher buoyancy in estuarine habitats include increased water content, larger size, lower dry weight, and thinner chorions with fewer lammellae [24], [25], [40], [43]. Experimental observations demonstrate that these traits are partly under osmotic responses but also have a population-level (adaptive) contribution. Moreover estuarine spawning populations have other reproductive adaptations for successful reproduction in these environments; these adaptations include fertilisation of eggs by sperm and egg development at lower salinities [83], [85], [86]. Successful spawning and reproduction by bluefin tuna would require similar adaptations.

Such adaptations are probably genetically based, although no study has yet to our knowledge identified the specific genes which might be responsible for these adaptations. Nevertheless, there is evidence that populations of several other marine fish species representing different taxa which spawn across wide salinity gradients are genetically distinct. For example, the physiological adaptations of cod reproduction in the eastern Baltic Sea [25], [26], [83], [85]–[87] where bottom salinity is 10–15 PSU may be associated with true genetic adaptations. Cod in the eastern Baltic Sea are sufficiently genetically different from other cod populations in the north Atlantic that the level of genetic variability from the North Sea through the Danish straits into the eastern Baltic Sea (a distance of only 600–800 km) is greater than the genetic variability throughout most of the entire latitudinal range in the northeast Atlantic [88]. This genetic adaptation must have occurred rapidly (in evolutionary terms) because cod are only believed to have invaded the Baltic Sea since the last glaciation ca. 10–15,000 years ago. Similar levels of genetic variability associated with the strong salinity gradient through the Danish straits have been documented for several other marine fish species (sprat, turbot, herring, flounder; [12]), several of which also produce eggs with density characteristics adapted for the estuarine conditions of the Baltic Sea.

There is evidence therefore from many other fish species across different taxa (e. g., Gadidae, Clupeidae, Pleuronectidae) that local populations can produce eggs whose specific gravities are adapted to local hydrographic conditions. Moreover, ichthyoplankton data show that other scombrid species (e. g., bonito *Sarda sarda* and Atlantic mackerel *Scomber scombrus*) and another large highly migratory top predator fish species (swordfish *Xiphias gladius*) also spawn in the Black Sea [67], [89], [90]. Given this comparative biogeographic evidence, we suspect therefore that at least some of the bluefin tuna which used to spawn in the Black Sea also were locally adapted to its particular hydrographic circumstances. The collection of eggs attributed as *T. thynnus* in this region and in the upper meters of the water column suggests that some bluefin tuna possessed these adaptations.

We also investigated the relative sizes of bluefin tuna eggs produced in habitats with different salinities. As is shown in the literature, estuarine-spawning populations usually produce larger eggs with higher water content than marine-spawning populations. The data available for bluefin tuna to evaluate this hypothesis are fragmentary and partly based on hormonally-induced spawning of adults fed artificial diets. It is unknown whether such treatment might affect buoyancy or sizes of eggs, if at all. Nevertheless, eggs collected in the Black Sea were among the largest of those which have been measured to date, although there is some overlap among measurements from different regions and populations. The egg size based evidence is therefore inconclusive, although not contradictory to the hypothesis that egg size was larger in the Black Sea than other areas.

### Recovery Possibilities for Black Sea Bluefin Tuna

As described in the literature, bluefin tuna have migrated to the Black Sea for millennia, but have recently disappeared [1], [3], [91]. If it indeed is true that spawning occurred there, and that the spawning was successful (i. e., capable of producing offspring that themselves survived to become new spawners), then we must assume that the population was adapted to produce eggs and larvae in that environment. This adaptation could have (1) been buoyancy – related (as is the case in estuarine spawning populations of many other marine species), (2) involved adaptation of the physiological requirements for temperature, salinity and oxygen concentration for egg and larval development, or (3) a combination of both types of adaptation. We note that even if the former Black Sea spawners could produce eggs whose buoyancy enabled them to float in warm oxic water, the eggs would have to be fertilized and develop at much lower salinities than in the Mediterranean Sea. However such adaptation may also have been possible because many other marine fish species have populations which can reproduce successfully in estuarine environments whose salinities are ca. 50–70% lower than in marine habitats [24], [40], [43], [92].

The recent demise of the Black Sea population component therefore probably represents a loss of intra-specific diversity, at least at the phenotypic level, and probably also at the genotypic level. Recent studies have highlighted the importance of population-level diversity in life histories and traits to sustain exploited populations, particularly under climate change and high levels of exploitation [16]–[18]. Loss of individual populations with locally-adapted traits (e. g., reproductive biology) increases the vulnerability of the species as a whole to further decline and possible extinction [9], [10]. The presence of distinct populations with different life histories and local adaptations is considered to increase species productivity and resilience to detrimental perturbations [18], [93].

In this study, we have developed from a comparative analysis of reproductive strategies in widely-spawning fish species and of oceanographic conditions, an hypothesis of the (former) existence of a locally-adapted Black Sea population. Should this hypothesis be correct, then spawning in the Black Sea by vagrant [9] bluefin tuna (i. e., those born in high-salinity areas) will not likely soon lead to recovery of a Black Sea population component, unless a substantial portion of their eggs can tolerate and be fertilized in low salinities and are sufficiently buoyant to avoid the deep, cold, low-oxygen layers of the Black Sea. As a result, the best chances for recovery of the Black Sea population are reproduction by locally-adapted individuals, if any of those are still remaining undetected. However, if this population has indeed been extirpated, another though admittedly slow recovery option is to continue and strengthen efforts to rebuild the populations which inhabit the Mediterranean Sea. These populations, which are managed collectively as part of the northeast Atlantic-Mediterranean stock, have declined in recent decades [94]–[96], but a larger population can via density-dependent processes be expected to increase the chances that vagrants could (re-)establish new spawning areas [9], [17], including in the Black Sea. This process could happen if Mediterranean abundances increased to levels which stimulated individuals to explore new territories for spawning and feeding. However we emphasize that reproductive success by any existing vagrant Mediterranean bluefin tuna in the Black Sea will not likely initially be high – reproductive success will probably require adaptation to the local conditions in this region. Recovery via this process will therefore be slow and require many generations.

Despite the apparent captures of eggs and larvae of bluefin tuna in the Black Sea, there has been some doubt expressed whether bluefin tuna ever did spawn in this region [62]. Bluefin tuna eggs are difficult to identify and distinguish from eggs of other species [1]. However the ichthyoplankton sampling and studies summarized here and in [1] were all part of extensive taxonomic descriptions of eggs and larvae in the region, and often included detailed descriptions of the eggs and larvae of many species. This suggests that the identifications were done by experts. In addition, we have shown using evidence from a wide variety of other marine species that it is common for such species to reproduce successfully in estuarine environments. We suspect that bluefin tuna may also have been such a species, but cannot eliminate the possibility of misidentification of earlier samplings. If spawning could definitively be confirmed or refuted (e. g., possibly through genetic analysis of existing eggs in collections, if any exist), then our ideas and hypotheses discussed here could also be confirmed or refuted.

If, alternatively, the migration to the Black Sea was for feeding, then a recovery or reappearance might occur sooner. Such a reappearance could occur if former feeding migration behaviours were re-established via for example exploratory (straying) foragers and social trans-generational learning of productive foraging areas by age and size-groups presently existing within the Mediterranean populations [17], [97]. These behaviours could be promoted both by recovery of the Mediterranean populations, which would motivate density-dependent exploration and discovery of new feeding habitats [17], [97], [98], and by recovery and maintenance of large forage fish populations within the Black Sea. Consequently management actions that could promote recovery in the Black Sea would be reduced exploitation of bluefin tuna throughout the Mediterranean and especially in the Aegean Sea-Sea of Marmara through which bluefin tuna must pass on the way to the Black Sea.

While the focus of this study is on the Black Sea and its tuna population, the study could be a precedent for what could happen to other species with locally adapted heavily exploited populations in estuarine situations. As noted earlier the eastern Baltic cod is adapted for reproduction in the low salinity conditions of the Baltic Sea; as with bluefin tuna in the Black Sea, its loss would not quickly be compensated by immigrants from neighboring higher saline waters like the Kattegat or North Sea because of population-specific differences in egg buoyancies and salinity tolerances [24], [26], [99].

### Roles of Eutrophication and Future Climate Change

Some of the problems associated with marine species spawning in the Black Sea could perhaps be related to the anoxic conditions in deeper layers where eggs and larvae might be neutrally buoyant. However this may not be the case for bluefin tuna. If bluefin tuna eggs were distributed in the upper few metres of the water column then they would experience high oxygen concentrations (Figure 2; S6). Oxygen conditions remain high, and comparable to those in other bluefin tuna spawning habitats around the world at least to 60 m depth, so even if the eggs were neutrally buoyant at such depths they should probably develop and hatch at rates independent of local oxygen concentrations. Indeed the more critical variables for egg survival in the Black Sea would appear to be temperature and salinity, given the limited knowledge of tolerances to these variables in the literature.

In this context, it is relevant to consider how future climate change might affect a possible recovery of spawning by bluefin tuna in the Black Sea. Although there are presently no local climate change projections for this ecosystem [100], a rise in temperature similar to that expected globally (ca. 2–4°C; [101]) and a regional decrease in precipitation or runoff should be beneficial for bluefin tuna spawning success in the Black Sea. Higher temperatures would benefit development rates and hatch success, whereas a reduction in precipitation might increase salinity and water column density. However, a full examination of the consequences of these impacts on a potential resumption of spawning by bluefin tuna in the Black Sea requires a climate change impact assessment for the region as well as new knowledge of bluefin tuna reproductive biology and early life history.

### Knowledge Gaps

While conducting our investigation, we became aware of several key knowledge gaps which if filled would help confirm or refute our ideas. Most fundamental would be a taxonomic and/or genetic re-analysis of any preserved eggs that may still be remaining in samples from the earlier collections to confirm or correct the previous identifications. New genetic technologies could resolve the issue with certainty, possibly even if samples are preserved in pH-buffered formalin [102]. A second major gap is knowledge of the buoyancy of eggs and larvae of Mediterranean *T. thynnus* in the laboratory and field. New and more extensive measurements would reveal part of the variability in buoyancy which could be the basis on which natural selection could act to regenerate a Black Sea spawning population. New experiments and field studies should be conducted to quantify the physiological thresholds of temperature, salinity and oxygen concentration which allow Mediterranean bluefin tuna eggs and larvae to be fertilized and develop successfully, and how these rates change with variations in female characteristics (size, age) and throughout the spawning season. These measurements would also be useful inputs not only for the kinds of analyses considered here, but also climate change impact studies and advanced coupled bio-physical modelling studies of early life history [28], [29], [103].

### Conclusions

We have used comparative approaches both within the bluefin tuna species and with other marine fish species which spawn across large salinity gradients to help understand the demise and historical ecology of bluefin tuna in the Black Sea. If there was a local successfully- reproducing population, it must have been adapted to the specific hydrographic conditions in this region. Mediterranean bluefin tuna could not likely produce eggs which could be fertilized and survive in the Black Sea without local adaptation, although we caution that the full plasticity of egg physiological responses to key abiotic variables has not been fully investigated and might allow survival. Evidence from many other species demonstrates the ecological potential for adaptation by marine fish species for spawning in estuarine habitats; given these other examples, it is possible that bluefin tuna may also have been adapted to spawn in such a habitat. This possibility is supported by several ichthyoplankton surveys which reportedly captured bluefin tuna eggs at times of year and at depths which would promote egg and larval survival. If locally – adapted Black Sea bluefin tuna spawners no longer exist, their recovery via evolutionary and natural selection processes will take many generations, based on vagrants from Mediterranean populations. Such vagrants may also lead to recovery in the Black Sea via re-establishment of former feeding migration behaviours. Notably, both mechanisms for reappearance of bluefin tuna in the Black Sea would occur faster if Mediterranean populations were larger.

## Supporting Information

Figure S1 a) Profiles for the long term and upper value density (σ_t_  =  density in kg m^−3^–1000) and (b) diffusivity (m s-2) profile at the beginning of the simulation and after 3 and 5 days. Note that numerical particles representing bluefin tuna eggs have been released at t0 = 3 days when the upper layers (<15 m depth) are well mixed due to the applied wind stress.
(TIF)

Figure S2 **Mean and range of diameters of bluefin tuna eggs from different geographic areas.** All eggs except those from the Black Sea were obtained from females held under semi-natural conditions. Data from the Tyrrhenian Sea are mean ± standard error instead of range. Data sources: [38], [56], [58], [60], [61].
(TIF)

Figure S3 **Vertical profiles of density (σ_t_  =  density in kg m** ^−**3**^ **–1000) in spawning areas for bluefin tunas around the world.** Solid and dashed lines are based on statistical fits using General Additive Modelling. Dots are observed data. See Table 1 for latitude – longitude coordinates and spawning times.
(TIF)

Figure S4 **Vertical profiles of salinity (absolute salinity g kg** ^−**1**^ **) in spawning areas for bluefin tunas around the world.** Solid and dashed lines are based on statistical fits using General Additive Modelling. Dots are observed data. See Table 1 for latitude – longitude coordinates and spawning times.
(TIF)

Figure S5 **Vertical profiles of temperature (°C) in spawning areas for bluefin tunas around the world.** Solid and dashed lines are based on statistical fits using General Additive Modelling. Dots are observed data. See Table 1 for latitude – longitude coordinates and spawning times.
(TIF)

Figure S6 **Vertical profiles of oxygen concentration (ml l** ^−**1**^ **) in spawning areas for bluefin tunas around the world.** Solid and dashed lines are based on statistical fits using General Additive Modelling. Dots are observed data. See Table 1 for latitude – longitude coordinates and spawning times.
(TIF)

Table S1 **Buoyancy of fish eggs measured in the field and in laboratory experiments for different species and populations within species as compiled from literature sources.** The table contains data for species with observations at multiple salinity levels. See main text for further details regarding data extraction and compilation. (Table available as spreadsheet from journal website.)
(XLSX)

Table S2 **Datasets used in statistical analyses and results of General Additive Modelling (GAM) of vertical distributions of temperature, salinity, density and oxygen concentration vs. depth in spawning areas of bluefin tunas in the global ocean.** All GAMs are statistically significant at P<0.0001. Abbreviations: N  =  sample size (number of years), Expl. Dev.  =  explained deviance, GCV  =  Generalized cross-validation statistic, EDF  =  estimated degrees of freedom.
(DOCX)
