# Supplementary figures and images for: Spawning of Bluefin Tuna in the Black Sea: Historical Evidence, Environmental Constraints and Population Plasticity
Source: PLoS One. 2012 Jul 24;7(7):e39998. doi: 10.1371/journal.pone.0039998 (PMC3404090; doi:10.1371/journal.pone.0039998)

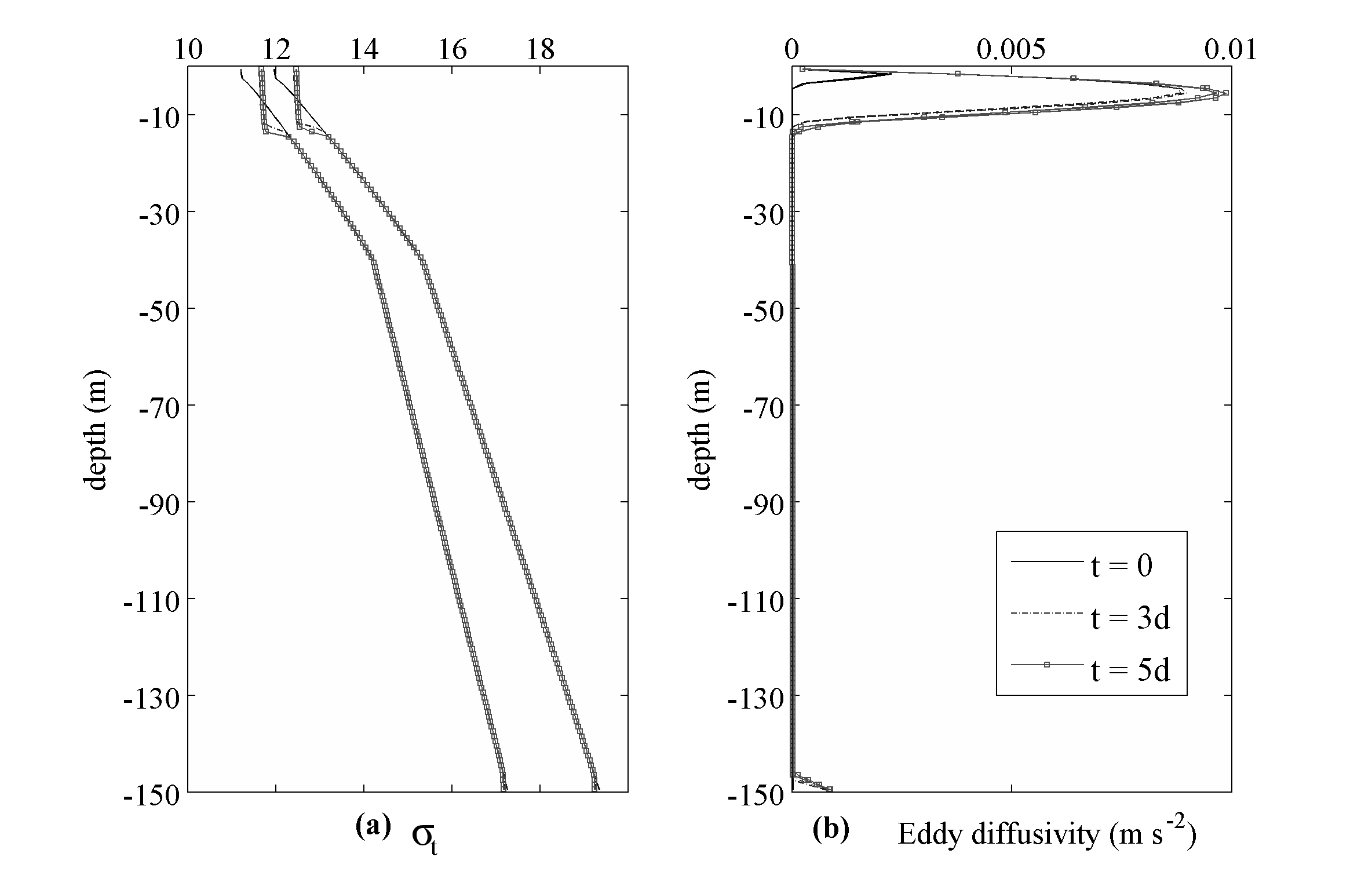

Supplement: Figure S1 — a) Profiles for the long term and upper value density (σt = density in kg m−3–1000) and (b) diffusivity (m s-2) profile at the beginning of the simulation and after 3 and 5 days. Note that numerical particles representing bluefin tuna eggs have been released at t0 = 3 days when the upper layers (<15 m depth) are well mixed due to the applied wind stress. (TIF) [file pone.0039998.s001.tif]

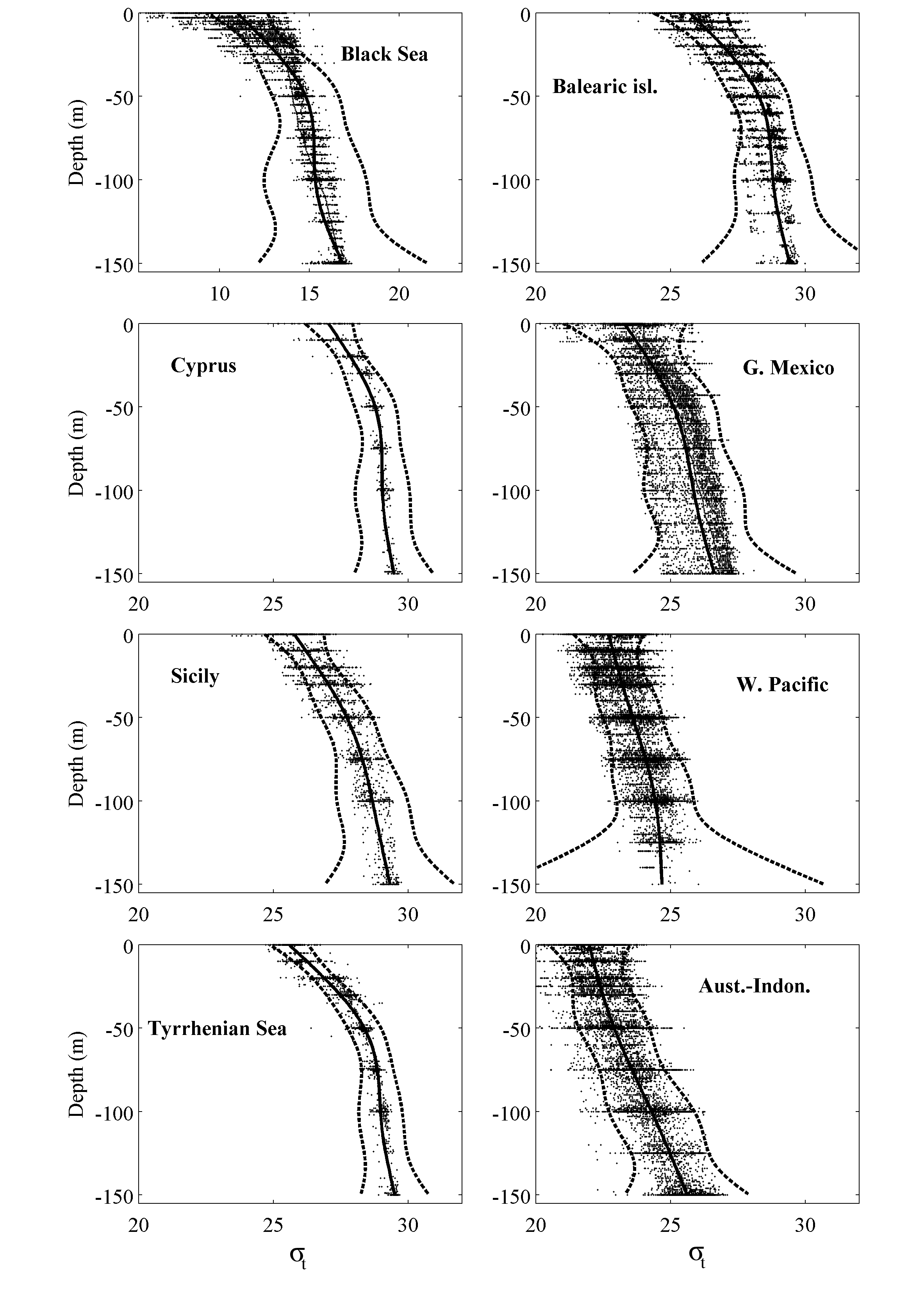

Supplement: Figure S3 — Vertical profiles of density (σt = density in kg m −3 –1000) in spawning areas for bluefin tunas around the world. Solid and dashed lines are based on statistical fits using General Additive Modelling. Dots are observed data. See Table 1 for latitude – longitude coordinates and spawning times. (TIF) [file pone.0039998.s003.tif]
