# Supplementary material for: Spawning of Bluefin Tuna in the Black Sea: Historical Evidence, Environmental Constraints and Population Plasticity
Source: PLoS One. 2012 Jul 24;7(7):e39998. doi: 10.1371/journal.pone.0039998 (PMC3404090; doi:10.1371/journal.pone.0039998)
Supplement: Table S2 — Datasets used in statistical analyses and results of General Additive Modelling (GAM) of vertical distributions of temperature, salinity, density and oxygen concentration vs. depth in spawning areas of bluefin tunas in the global ocean. All GAMs are statistically significant at P<0.0001. Abbreviations: N = sample size (number of years), Expl. Dev. = explained deviance, GCV = Generalized cross-validation statistic, EDF = estimated degrees of freedom. (DOCX) [file pone.0039998.s008.docx]

Spawning of Bluefin Tuna in the Black Sea: Historical Evidence, Environmental Constraints and Population Plasticity

Brian R. MacKenzie^1*^ and Patrizio Mariani^2^

^1^Center for Macroecology, Evolution and Climate

National Institute for Aquatic Resources (DTU Aqua)

Technical University of Denmark

Charlottenlund

Denmark

^2^Center for Ocean Life

National Institute for Aquatic Resources (DTU Aqua)

Technical University of Denmark

Charlottenlund

Denmark

^*^corresponding author: [brm@aqua.dtu.dk](mailto:brm@aqua.dtu.dk)

Supplementary Table S2. Datasets used in statistical analyses and results of General Additive Modelling (GAM) of vertical distributions of temperature, salinity, density and oxygen concentration vs. depth in spawning areas of bluefin tunas in the global ocean. All GAMs are statistically significant at P < 0.0001. Abbreviations: N = sample size (number of years), Expl. Dev. = explained deviance, GCV = Generalized cross-validation statistic, EDF = estimated degrees of freedom.

| Region | Variable | Years | N | Expl. Dev. | GCV | EDF |
| --- | --- | --- | --- | --- | --- | --- |
| Black Sea | density | 1890-2001 | 13861 | 0.77 | 0.91 | 3 |
| Balearic isl. |  | 1910-1998 | 5714 | 0.77 | 0.39 | 3 |
| Cyprus |  | 1962-1981 | 679 | 0.78 | 0.17 | 3 |
| G. Mexico |  | 1932-1995 | 7675 | 0.56 | 0.78 | 3 |
| Sicily |  | 1880-1998 | 2469 | 0.8 | 0.29 | 3 |
| W. Pacific |  | 1925-2005 | 16549 | 0.49 | 0.4 | 3 |
| Tyrrhenian Sea |  | 1880-1996 | 1675 | 0.94 | 0.12 | 3 |
| Aust.-Indon. |  | 1916-2004 | 13204 | 0.73 | 0.5 | 3 |
| Black Sea | temperature | 1890-2001 | 13861 | 0.77 | 9.43 | 7 |
| Balearic isl. |  | 1910-1998 | 5714 | 0.81 | 2.68 | 7 |
| Cyprus |  | 1962-1981 | 679 | 0.75 | 1.54 | 5 |
| G. Mexico |  | 1932-1995 | 7675 | 0.4 | 7.39 | 6 |
| Sicily |  | 1880-1998 | 2469 | 0.73 | 3.13 | 6 |
| W. Pacific |  | 1925-2005 | 16549 | 0.32 | 3.81 | 5 |
| Tyrrhenian Sea |  | 1880-1996 | 1675 | 0.92 | 1.28 | 7 |
| Aust.-Indon. |  | 1916-2004 | 13204 | 0.72 | 3.71 | 6 |
| Black Sea | salinity | 1890-2001 | 13861 | 0.55 | 0.59 | 3 |
| Balearic isl. |  | 1910-1998 | 5714 | 0.17 | 0.16 | 3 |
| Cyprus |  | 1962-1981 | 679 | 0.05 | 0.06 | 3 |
| G. Mexico |  | 1932-1995 | 7675 | 0.14 | 0.39 | 3 |
| Sicily |  | 1880-1998 | 2469 | 0.42 | 0.11 | 3 |
| W. Pacific |  | 1925-2005 | 16549 | 0.13 | 0.05 | 3 |
| Tyrrhenian Sea |  | 1880-1996 | 1675 | 0.58 | 0.04 | 3 |
| Aust.-Indon. |  | 1916-2004 | 13204 | 0.04 | 0.24 | 3 |
| Black Sea | oxygen | 1924-1998 | 4484 | 0.68 | 1.42 | 6 |
| Balearic isl. |  | 1910-1997 | 2189 | 0.37 | 0.13 | 7 |
| Cyprus |  | 1966-1977 | 417 | 0.04 | 0.47 | 3 |
| G. Mexico |  | 1935-1995 | 1737 | 0.44 | 0.26 | 4 |
| Sicily |  | 1910-1998 | 495 | 0.28 | 0.11 | 4 |
| W. Pacific |  | 1929-2005 | 5723 | 0.03 | 0.09 | 5 |
| Tyrrhenian Sea |  | 1910-1995 | 342 | 0.58 | 0.1 | 6 |
| Aust.-Indon. |  | 1929-2004 | 4982 | 0.6 | 0.19 | 5 |
